# Surfing the web as a patient with IBD: New horizons

**DOI:** 10.1002/ueg2.12437

**Published:** 2023-07-06

**Authors:** Iago Rodríguez‐Lago, Ignacio Catalan‐Serra, Manuel Barreiro‐de Acosta

**Affiliations:** ^1^ Gastroenterology Department Hospital Universitario de Galdakao Biocruces Bizkaia Health Research Institute Galdakao Spain; ^2^ School of Medicine University of Deusto Bilbao Spain; ^3^ Gastroenterology Department Levanger Hospital Nord‐Trøndelag Hospital Trust Trondheim Norway; ^4^ Department of Molecular Medicine Centre of Molecular Inflammation Research (CEMIR) Norwegian University of Science and Technology (NTNU) Trondheim Norway; ^5^ Gastroenterology Department Hospital Clínico Universitario de Santiago Santiago de Compostela Spain

**Keywords:** Crohn's disease, IBD, inflammation, inflammatory bowel disease, ulcerative colitis

Inflammatory bowel disease (IBD) is a chronic lifelong condition with a disabling course in a significant proportion of patients. Additionally, it is more frequently diagnosed during childhood and in young adults, so its impact can be observed in many aspects of their life in the long‐term.^1^ Despite this negative perspective, the therapeutic armamentarium has increased in recent years, so the probability of controlling the disease activity has increased notably.

As we continue to learn about the triggers of the disease and the underlying immunological alterations, our understanding of the real impact of this condition is also growing. For instance, it is now clear how the disease may affect extraintestinal organs^2^ but, even more importantly, also the critical impairment of quality of life, disability, mental health and work productivity.^3^ This is highly relevant, since this impact on several domains of the patients' lives might be present despite an adequate control of the disease in the gut. The increasing awareness of its impact led the European Medicines Agency (EMA) to express their support to include quality of life and patient‐reported outcomes (PRO) in IBD clinical trials in 2019.^4^ Crohn's disease (CD) and ulcerative colitis‐PRO signs and symptoms diaries were developed and endorsed by EMA for this purpose,^5^, ^6^ and they are now considered as the first choice in the assessment of disease activity as the use of other scores like Crohn's Disease Activity Index and total Mayo are difficult to use in daily practice or can be biased in certain situations. These new endpoints and trial designs have already been implemented during the development of the most recently developed biologics and small molecules^7^, ^8^ and hopefully can lead to broader the concept of disease‐modification strategies in the near future.^9^

Thus, the shift in the management of these patients has evolved from a physician‐centered model to a patient‐focused evaluation. This is expected to bring new perspectives with more realistic and impactful unmet needs, and they can also potentially reduce patient‐care variability.^10^ Despite this remarkable ‐and positive‐ change, it should be stressed that both perspectives (from physicians and patients) have demonstrated significant differences in the assessment of disease activity or adverse drug reactions.^11^, ^12^ Nevertheless, in the era of electronic health records, digital PRO have been developed in parallel with telemedicine resources; thus, patient wellbeing, certain follow‐up consultations and patient self‐management are new tools available to improve a more comprehensive evaluation of the disease.^13^ We should all be involved in implementing these advances within our clinical practice, as this is a unique opportunity to work together and understand what patients need more in detail.

Platforms where patients can report their perception of the disease and interact with their physicians are urgently needed. Ideally, they should also serve as a source for research that can explore new treatment alternatives and how to fulfil other patient needs. However, new tools also bring new questions.^14^ Digital platforms, and mainly those using cell phones, may not be suitable for all patients and across all ages. Accessibility will always be an issue, as they need to encourage the participation of those (not so) motivated patients, those with disabilities, and those without limited access to certain technologies.

In this issue, Engel et al. provide new and useful information about how these resources can be applied for research.^15^ Here, StuffThatWorks (www.stuffthatworks.health), a platform designed to collect clinical information from chronic conditions using a machine learning algorithm, was used to gather patient experience on different medical treatments for CD. The authors showed that anti‐TNF agents were ranked as the most effective by patients, followed by ustekinumab and vedolizumab, respectively. Interestingly, surgery appeared in second place. However, one of the most remarkable aspects evaluated in the study is the triggers associated with disease activity, highlighting that psychological factors and certain foods were the most important factors reported by patients. Nonetheless, we also think that validation across diseases and countries would increase the value of their interesting findings.

In conclusion, the featured paper by Israeli colleagues provides novel and relevant information about how new technologies can bridge the gap between patient and physician perceptions in IBD and to include PRO in future research. Still, some questions remain open about how these data could be combined with traditional trials. Nonetheless, in our opinion, these novel digital approaches explore how we can closely collaborate with patients in order to shape and improve the future clinical care of patients affected with IBD.

## CONFLICT OF INTEREST STATEMENT

IR‐L has received financial support for travelling and educational activities from or has served as an advisory board member for Abbvie, Adacyte, Celltrion, Chiesi, Danone, Ferring, Faes Farma, Janssen, Galapagos, MSD, Pfizer, Roche, Takeda, and Tillotts Pharma. Financial support for research: Tillotts Pharma. IC‐S has received financial support for travelling and educational activities from or has served as an advisory board member for Takeda, AbbVie, Janssen, Tillots Pharma, Ferring, Lilly, and BMS. MB‐A has received financial support for travelling and educational activities from or has served as an advisory board member for Pfizer, MSD, Takeda, AbbVie, Kern, Janssen, Fresenius Kabi, Galapagos, Lilly, BMS, Faes Farma, Chiesi, and Adacyte.

## FUNDING INFORMATION

Biocruces Bizkaia Health Research Institute, Grant/Award Number: BCB/I/LIB/22/008; Gobierno Vasco – Eusko Jaurlaritza, Grant/Award Number: 2020111061

## Data Availability

Not applicable.
